# Acute administration of roflumilast enhances sensory gating in healthy young humans in a randomized trial

**DOI:** 10.1007/s00213-017-4770-y

**Published:** 2017-11-03

**Authors:** Pim R. A. Heckman, Marlies A. Van Duinen, Arjan Blokland, Tolga Uz, Jos Prickaerts, Anke Sambeth

**Affiliations:** 1 Department of Psychiatry and Neuropsychology, School for Mental Health and Neuroscience, Maastricht University, 6200 MD Maastricht, The Netherlands; 2 Department of Neuropsychology and Psychopharmacology, Maastricht University, PO Box 616, 6200 MD Maastricht, The Netherlands; 3 Experimental Medicine CNS, Takeda Development Center Americas, Inc., Deerfield, MA USA

**Keywords:** Phosphodiesterase, Phosphodiesterase inhibitor, Roflumilast, Sensory gating, Clinical trial

## Abstract

**Introduction:**

Sensory gating is a process involved in early information processing which prevents overstimulation of higher cortical areas by filtering sensory information. Research has shown that the process of sensory gating is disrupted in patients suffering from clinical disorders including attention deficit hyper activity disorder, schizophrenia, and Alzheimer’s disease. Phosphodiesterase (PDE) inhibitors have received an increased interest as a tool to improve cognitive performance in both animals and man, including sensory gating.

**Methods:**

The current study investigated the effects of the PDE4 inhibitor roflumilast in a sensory gating paradigm in 20 healthy young human volunteers (age range 18–30 years). We applied a placebo-controlled randomized cross-over design and tested three doses (100, 300, 1000 μg).

**Results:**

Results show that roflumilast improves sensory gating in healthy young human volunteers only at the 100-μg dose. The effective dose of 100 μg is five times lower than the clinically approved dose for the treatment of acute exacerbations in chronic obstructive pulmonary disease (COPD). No side-effects, such as nausea and emesis, were observed at this dose. This means roflumilast shows a beneficial effect on gating at a dose that had no adverse effects reported following single-dose administration in the present study.

**Conclusion:**

The PDE4 inhibitor roflumilast has a favorable side-effect profile at a cognitively effective dose and could be considered as a treatment in disorders affected by disrupted sensory gating.

## Introduction

Sensory gating is a process involved in early information processing which prevents overstimulation of higher cortical areas by filtering sensory information (Freedman et al. 1983). The typical sensory gating paradigm consists of two identical auditory stimuli that are presented shortly after each other. The main principle is that the response, as measured by scalp EEG, to the second stimulus (S2) will be smaller than the response to the first stimulus (S1). In humans, the P50 (i.e., the response evoked 50 ms after stimulus onset) of the event-related potential (ERP) is believed to be the main component in the sensory gating paradigm. Although the P50 reflects information processing at early stages, it has also been associated with different cognitive functions (Yadon et al. 2009).

Human research has shown indications for disruptions in sensory gating in clinical disorders including attention deficit hyperactivity disorder (ADHD), schizophrenia, and Alzheimer’s disease (Adler et al. 1982; Ally et al. 2006; Micoulaud-Franchi et al. 2015). However, the whole concept of cognition in humans and the relation to sensory gating is still under investigation. For instance, the P50 has been suggested as a biomarker for the evaluation of drugs that may potentially have a beneficial effect on cognitive functions in schizophrenia (Javitt et al. 2008), but despite the prominent role that P50 abnormalities have played in our understanding of schizophrenia, more data is needed to fully incorporate P50 as clinical correlate (Potter et al. 2006). One advantage of this EEG-related measure is that it can be used for translational purposes (Blokland et al. 2015).

**Fig. 1:**
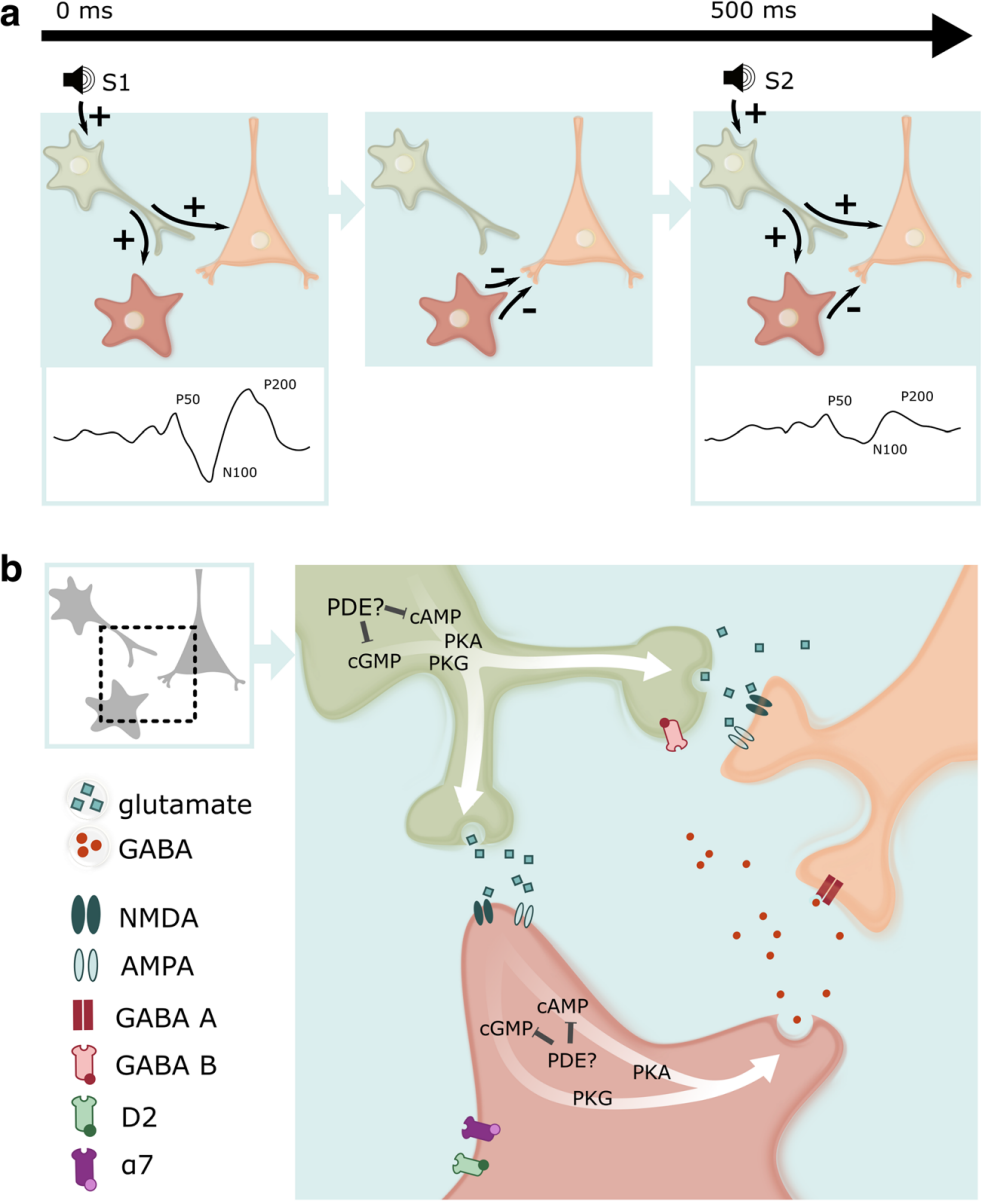
Effects of PDE inhibitors on sensory gating are believed to be induced by targeting PDEs expressed in inhibitory interneurons of the auditory cortex and the thalamic “gate,” frontal inhibitory output neurons or in the interneurons that locally release inhibitory neurotransmitter in any other brain area capable of eliciting sensory gating. **a** Auditory stimulus 1 (S1) excites an excitatory neuron, which in turn excites an inhibitory interneuron as well as an excitatory pyramidal neuron (left side figure). Activation of the inhibitory interneuron induces release of the inhibitory neurotransmitter GABA. GABA release causes fast inhibition of the pyramidal neuron via postsynaptic GABA-A receptors (middle figure). Additionally, GABA released from the inhibitory interneurons induces slow, persistent inhibition of glutamate release from the first excitatory neuron onto the pyramidal neuron via presynaptic GABA-B receptors. This persistent inhibition reduces the activity of the pyramidal neuron for up to 8 s. Consequently, if S2 arrives, the ERP amplitude will be reduced (right side figure). **b** More detailed depiction of the processes explained in **a**, showing the release site of glutamate and GABA as well as the location of their respective receptors. The postsynaptic GABA-A receptor inhibits the pyramidal neuron after activation by S1. The presynaptic GABA-B receptor induces the persistent inhibition of the first excitatory neuron thereby inducing gating. Targeting any PDE subtype, e.g., PDE4, expressed in the inhibitory interneuron itself could enhance GABA release from the inhibitory interneuron when activated by S1. Additionally, any PDE subtype, e.g., PDE4, expressed in the first excitatory neuron’s projections to the inhibitory interneuron could also enhance GABA release in the inhibitory interneuron. Consequently, both will result in an enhanced reduction of the S2-induced ERP amplitude. Note, however, that in the latter case, when the particular PDE subtype is expressed in the first excitatory neuron’s projections to the inhibitory interneuron, this might also result in an enhanced response to S1. However, the latter is not observed in our study, indicating that the effect of roflumilast is more likely to occur in the inhibitory interneurons themselves. Finally, the dopamine D2 receptor as well as the cholinergic α7 nicotinic receptor is depicted on the inhibitory interneuron. It is known from other studies that antipsychotic medication (D2 antagonists) enhances sensory gating. This possibly occurs via antagonism of the inhibitory effect D2 receptors exerted on cAMP signaling and subsequent GABA release. In a similar but opposite manner, activating α7 nicotinic receptors on inhibitory interneurons enhances cAMP signaling in these neurons and increases associated GABA release

In the last decades, phosphodiesterase (PDE) inhibitors have received an increasing interest as a tool to improve cognitive performance in both animals and man (Reneerkens et al. 2009). As the initial focus of cognition enhancement was directed towards memory function, nowadays the relation between PDEs and cognitive processing is also investigated beyond the memory domain (e.g., Heckman et al. 2016). One cognitive process in which PDE inhibitors might play a role is sensory gating (see Fig. 1).

Overall, only a limited number of human and animal studies have tested the effects of PDE inhibitors on sensory gating. Redrobe and colleagues (2014), using the relatively new PDE2 inhibitor Lu AF64280, managed to induce an effect on sensory gating, i.e., reduction of an amphetamine-induced gating deficit in DBA2 mice. Two studies by Reneerkens et al. (2013a, b) found no effects of either the PDE2 inhibitor BAY 60-7550 in rats, or the PDE5 inhibitor vardenafil in healthy rats as well as healthy young humans (in the absence of a deficit model). Another class of PDE inhibitors tested in the sensory gating paradigm is PDE10A inhibitors. PDE10A inhibitors are chosen with respect to the search for new antipsychotics in the field of schizophrenia research due to the high and exclusive expression of PDE10A in the striatum (Lakics et al. 2010). However, the effects of PDE10A inhibitors in animal models are mixed. On the one hand, no effects were found for the PDE10A inhibitor PQ-10 in healthy rats (Reneerkens et al. 2013a), an amphetamine-deficit model, or a phencyclidine (PCP) deficit model (Ahnaou et al. 2016). On the other hand, TP-10, another more potent PDE10A inhibitor, reversed impaired sensory gating in the hippocampus using the amphetamine-induced deficit model in rats (Schmidt et al. 2008).

The PDE9 inhibitor PF-4447943 and PF-4449613 reversed an amphetamine-induced sensory gating deficit in mice (Kleiman et al. 2012). Additionally, PF-4447943 was tested in transgenic BACHD rats and Q175 mice (both transgenic animal models for Huntington’s disease exhibiting impaired sensory gating) (Nagy et al. 2015). PF-4447943 dose-dependently improved the gating deficit in the primary auditory cortex and hippocampus of transgenic BACHD rats. Daily administration of PF-04447943 (1 mg/kg) over 7 days resulted in a complete recovery in their auditory gating in two brain regions (i.e., the cortex and hippocampus). In Q175 mice, including wild-type, heterozygote, and homozygote mice, PDE9 inhibition was without any effect. PDE4 inhibitors were tested in a sensory gating paradigm in two separate studies. The first study tested the first-generation PDE4 inhibitor rolipram (Maxwell et al. 2004) and found that rolipram normalized the amphetamine-induced gating deficit in the hippocampus of mice. Another PDE4 inhibitor, RO-20-1724, (Halene and Siegel 2008) also restored gating in the hippocampus of mice using an amphetamine-induced deficit model.

Although there are great similarities between the ERPs of humans and rats, it has been argued that basic components of sensory gating may still differ between both species (e.g., de Bruin et al. 2001). Nevertheless, compared to other cognitive measures, ERPs are one of the most translational tools available to date (Blokland et al. 2015). Therefore, we expect that the effects of PDE inhibitors on sensory gating in animals translate to humans (c.f., Maxwell et al. 2004). Based on the few studies that are available, it appears that PDE4 is a promising PDE subtype in this respect (see Heckman et al. 2015). The PDE4 inhibitor roflumilast was the first oral-obtainable PDE4 inhibitor clinically approved at a daily dose of 500 μg, i.e., to treat chronic obstructive pulmonary disease (COPD). However, some typical side-effects have been observed for PDE4 inhibitors, such as nausea and emesis. Recently, we have shown that roflumilast is a brain penetrant and improves short-term and long-term memory in rodents (Vanmierlo et al. 2016). Importantly, a PET study with the ligand [18F]B9302-107 for roflumilast confirmed that the currently marketed dose for COPD is also a brain penetrant in humans (http://www.accessdata.fda.gov/drugsatfda_docs/nda/2011/022522Orig1s000ClinPharmR.pdf, p. 150–151). On basis of this existing data, we investigated the effects of the PDE4 inhibitor roflumilast on sensory gating in humans. We tested the acute effects in healthy young human volunteers at three different doses in a double-blind placebo-controlled study. We hypothesized roflumilast to enhance sensory gating in healthy young humans without exhibiting an effect on overall auditory processing as indicated by auditory-evoked potentials (AEP).

## Methods

### Participants

All experimental procedures were approved by the independent Ethics Committee of Maastricht University and the Academic Hospital Maastricht (The Netherlands). The study was conducted according to the code of ethics on human experimentation established by the Declaration of Helsinki (1964) and amended in Edinburgh (2000) and in accordance with the Medical Research Involving Human Subjects Act (WMO). The participants (age range 18–35 years; mean age 20.9 ± 2.3 years; 4 males/16 females) were recruited through advertisements at Maastricht University between November 2011 and June 2012. Participants had to be willing to sign an informed consent form and were paid for their participation. The subjects’ physical and mental health was checked by a physician (forensic doctor) by means of a standard medical questionnaire, including psychological and psychiatric evaluation, and a medical examination. Subjects were excluded if they suffered from or had a history of cardiac, hepatic, renal, pulmonary, neurological, gastrointestinal, hematological, or psychiatric illness. Other exclusion criteria were excessive drinking (> 20 glasses of alcohol-containing beverages a week), pregnancy or lactation, use of medication other than oral contraceptives, use of recreational drugs from 2 weeks before and until the end of the experiment, and any sensory or motor deficit which could reasonably be expected to affect test performance. In addition, participants who had a first-degree relative with a (history of) psychiatric disorder were excluded as well. The participants could leave the study at any given time without any consequences. All participants provided written informed consent after receiving a complete description of the study.

### EEG recordings

An EEG cap was used to place a set of 32 EEG electrodes according to the international 10–20 system (Klem et al. 1999). Only the Fz, Fcz, and Cz locations were used in the current study since it has been demonstrated previously that midline electrodes show better P50 sensory gating than left/right hemispheric sites (Wan et al. 2006). In addition, the Fz electrode has been demonstrated to show a similar amount of P200 gating (Wan et al. 2007). A reference and a ground were placed at the left mastoid and at the forehead, respectively. Eye movements were detected by horizontal and vertical electro-oculogram (EOG) recordings. Before electrode attachment, the positions were slightly scrubbed with a gel in order to provide a good measurement. Both EEG and EOG were filtered between 0.01 and 100 Hz and sampled at 1000 Hz. The sensory gating paradigm consisted of 60 pairs of identical auditory stimuli with a duration of 3 ms and intensity of 80 dB. Since testing took place in a sound attenuated room with a maximal background noise level of 20 dB, the stimulus salience was approximately 60 dB. The interval between the first (S1) and the second (S2) stimulus was 500 ms; the interval between pairs was randomized between 6 and 10 s. The participants were familiarized with the test during a training session.

### Design and treatment

The study was conducted according to a double-blind, placebo-controlled, four-way cross-over design. The current study was part of a larger project, investigating the cognition-enhancing effects of roflumilast. The treatment order was balanced over the four test days and separated by a washout period of at least 10 days. The balancing of the treatment order was accomplished by using a Latin square design. Roflumilast HCl (Daxas) 500-μg tablets were grinded, and the appropriate quantities (i.e., 100, 300, 1000 μg) were distributed over capsules with lactose monohydrate as the principle constituent. The placebo capsules only contained lactose monohydrate in an equivalent amount and the appearance was identical to the roflumilast capsules. The capsules were manufactured, blinded, and labeled by Basic Pharma Technologies BV (Geleen, the Netherlands) according to GMP regulations. Randomization personnel (not otherwise involved in the study) generated the randomization schedule, which was provided to the contract packaging facility prior to the start of the study. All randomization information was stored in a secured area, accessible only by authorized personnel. Treatment on each of the four test days consisted of a single capsule containing either placebo, 100, 300, or 1000 μg roflumilast. Previous studies have shown that peak plasma levels of roflumilast were reached 30–120 min (median, 60 min) after a single dose of 500 μg roflumilast; the terminal half-life was around 17 h for roflumilast and 30 h for its N-oxide metabolite (Bethke et al. 2007). The sensory gating paradigm was tested 90 min after drug administration. The drugs were ingested orally and combined with a low-fat breakfast, because fatty food might affect the absorption of roflumilast. Blood samples were taken 135 min after drug administration to determine plasma levels of roflumilast. The experimenter and participants were blind to the compound and doses tested. All testing was conducted at the department of Neuropsychology and Psychopharmacology at Maastricht University.

### Questionnaire

After each session, the subjects were asked to fill in a questionnaire. Physical complaints were measured by a general list consisting of 31 items with a 4-point scale ranging from 0: ‘not at all” to 3: “strongly.”

### Statistical analysis

All EEG data was analyzed with Vision Analyzer 2.0 (Brain Products GmbH, Gilching, Germany). After offline re-referencing the signal combining the left and right mastoids, the EEG signal was filtered with a high-pass filter of 10 Hz and a low-pass filter of 40 Hz. Next, eye movement artifacts were removed using the Gratton and Coles method (Gratton et al. 1983). Segments between 100 ms before and until 500 ms after stimulus onset were constructed for each stimulus type (S1 and S2) separately, using the last 100 ms before S1 onset as baseline for both stimuli. The segments were visually checked for artifacts and removed from the dataset if an artifact occurred during the first 500 ms after stimulus presentation. The grand average over participants was used to determine the AEP components. P50 was defined as the most positive value between 65 and 110 ms after stimulus onset, N100 as the most negative value between 90 and 170 ms and P200 as the most positive value between 170 and 260 ms. Due to violation of normality, data was analyzed using nonparametric tests for the amplitudes of the AEP component at the Fz, FCz, and Cz locations (channels). First, outliers were removed from the raw data. Next, effects of roflumilast on basal information processing were evaluated by comparing treatment effects on auditory-evoked potentials (S1). Subsequently, the responses to the S1 and S2 (S1-S2/S2) were compared by means of Wilcoxon Signed-ranks tests for the placebo condition to determine whether sensory gating occurred. Next, the responses to the roflumilast conditions were compared with those of the placebo condition for the scores by means of Wilcoxon Signed-ranks tests (IBM SPSS Statistics 24 software; IBM, Portsmouth, UK).

## Results

### Plasma levels

In the current study, the three doses of roflumilast administered resulted in plasma levels at the time of testing which could be expected based on PK studies measuring roflumilast, 2.09, 6.27, and 8.19 ng/mL, respectively (Lahu et al. 2011).

### Physical complaints

After administration of 100 μg, the subjects did not report any physical complaint. At 300 μg, four subjects reported mild nausea, but no other complaints. After the highest dose, mild nausea was reported by three subjects and five subjects reported a higher level of nausea. In addition, two subjects reported diarrhea at the highest dose.

### Effects of roflumilast on auditory-evoked potentials (S1 or S2)

No effects of PDE4 inhibition by roflumilast (100, 300, and 1000 μg) compared to placebo treatment were found with the Wilcoxon Signed-ranks test on the S1 or S2 stimulus for the P50 peak for neither the Fz channel nor the FCz and Cz channels (data not shown). Also, no effects were found on the two other ERP components (N100 and P200; data not shown).

### The sensory gating paradigm

**Fig. 2:**
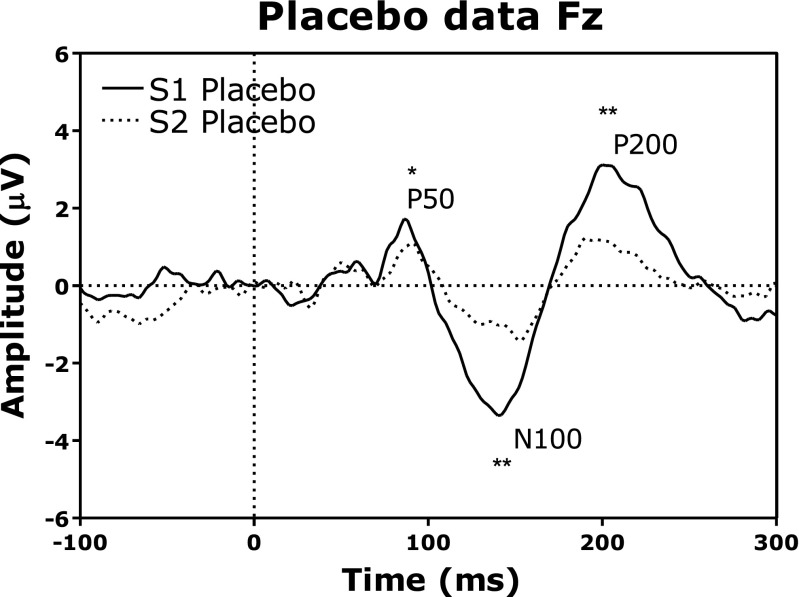
Placebo ERPs (P50, N100, and P200 peaks) after presentation of S1 and S2. Sensory gating, i.e., a difference between S1 and S2, is depicted with *asterisks* (Wilcoxon Signed-ranks test: *p < .05; ***p < .001). Latencies are shown on the x-axis in milliseconds, amplitudes on the y-axis in microvolts

Effects of placebo treatment on sensory gating for the Fz electrode are depicted in Fig. 2. Analysis by means of a Wilcoxon Signed-ranks test for the three channels Fz, FCz, and Cz separately showed that gating occurred in all three channels for the P50 peak (*Z* = − 2.05, *p* < .05; *Z* = − 3.10, *p* < .01; *Z* = − 2.91, *p* < .01). Additionally, sensory gating occurred for the N100 peak for all three channels (*Z* = − 3.54, *p* < .001; *Z* = − 3.88, *p* < .001; *Z* = − 3.85, *p* < .001) as well as for the P200 peak of all three channels (*Z* = − 3.68, *p* < .001; *Z* = − 3.92, *p* < .001; *Z* = − 3.82, *p* < .001).

### Effects of roflumilast on sensory gating

**Fig. 3:**
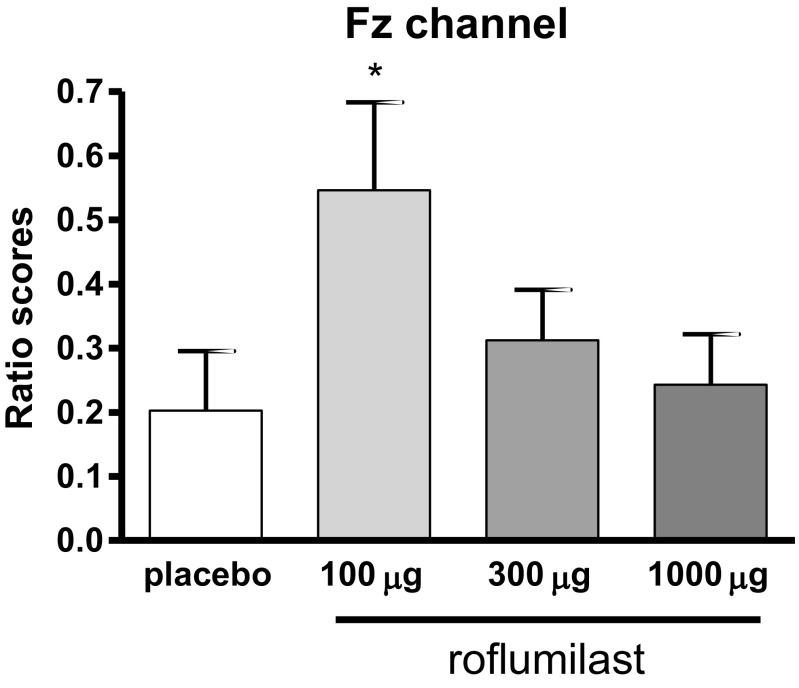
Effects of treatment with the PDE4 inhibitor roflumilast on the mean relative gating score (± SEM) of the P50 peak of the Fz channel. An effect on sensory gating, i.e., different gating scores compared to placebo, is depicted with an *asterisk* (Wilcoxon Signed-ranks test: *p < .05). Compounds/doses are depicted on the x-axis; ratio scores are depicted on the y-axis (higher ratio scores indicate better sensory gating)

The effect of roflumilast (100, 300, and 1000 μg) on sensory gating is shown in Fig. 3. A Wilcoxon Signed-ranks test indicated that sensory gating significantly improved at the Fz electrode for the P50 component after treatment with 100 μg roflumilast compared to placebo, *Z* = − 2.01, *p* < .05. No effects of roflumilast are found on the N100 or P200 ERP components of any of the channels.

## Discussion

In the current study, we investigated whether the PDE4 inhibitor roflumilast could enhance sensory gating in healthy young human volunteers without exhibiting an effect on overall auditory processing as indicated by AEP. Results showed that roflumilast significantly improved sensory gating in healthy young human volunteers in a dose-dependent manner. The effective dose of 100 μg is five times lower than the clinically approved dose for the treatment of acute exacerbations in COPD. Notably, no emetic side-effects were reported by the participants after administration of this low dose. This means roflumilast shows a beneficial effect on gating at a dose that had no adverse effects reported following single-dose administration in the present study. Nausea was only occasionally reported at the 300- and 1000-μg dose. This shows a favorable side-effect profile of roflumilast at a dose of 100 μg.

As shortly mentioned before, a clear distinction should be made between effects on AEPs (S1) and effects on sensory gating, even though both are considered “early information processing.” Different PDE families and their inhibitors can distinctively affect AEPs and sensory gating. Furthermore, whether sensory gating is expressed as a ratio score (e.g., S2/S1), difference score (e.g., S1 − S2), proportional score (e.g., S1 − S2/S2), or percentage score (e.g., (S1 − S2/S2)× 100), it always explains S2 in terms of S1. An effect on AEPs after S1 will also change the ratio between S1 and S2 which has to be taken into consideration when interpreting an effect on sensory gating. An effect on S1 indicates an effect on basic information processing. To induce a true effect on sensory gating, S1 should not be affected by the drug. A significant S2 effect (decreasing amplitude) would support drug effects on sensory gating. However, this is not necessary, as long as the relative gating score is showing significant drug effects, i.e., there is a difference on this score between drug conditions. We found that S1 did not differ between the placebo and the 100 μg roflumilast condition. Also, roflumilast did not affect S1 and that the S1-S2 ratio was enhanced after treatment with the 100-μg dose. This indicates that roflumilast specifically enhances P50 gating in young healthy volunteers.

Another point of attention regards the fact that in preclinical studies, an amphetamine-induced deficit was reversed by a PDE4 inhibitor (Maxwell et al. 2004; Halene and Siegel 2008). This might be related to a similar mechanism compared to enhanced unimpaired sensory gating in healthy volunteers. In schizophrenia, the dopamine hypothesis has been revised to postulate that positive symptoms, in particular, arise from hyperactivation of the dopaminergic D2 receptor subtype in mesolimbic brain regions (Brisch et al. 2014). Disruptive effects of amphetamine on sensory gating are suggested to be caused by hyperactive dopamine transmission resembling the dopamine hypothesis in schizophrenia (Smucny et al. 2015). Thus, amphetamine increases the levels of mesolimbic dopamine and this extra dopamine activates the mesolimbic D2 receptors on the inhibitory interneurons in, for instance, the hippocampus. Activation of D2 receptors inhibits the inhibitory interneurons. Excessive dopamine levels will thus lead to excessive throughput and thereby impair normal gating. This hypothesis is supported by the fact that D2 receptor antagonists can prevent the amphetamine-induced deficits in sensory gating (During et al. 2014; Witten et al. 2016). D2 receptor antagonism prevents inhibition of the inhibitory interneurons responsible for sensory gating by amphetamine.

However, it should be noted that in the field of schizophrenia research, dopaminergic drugs (D2 antagonists) generally show no gating-enhancing effects. Also, D2 receptor antagonism has not convincingly shown to affect sensory gating in healthy subjects (either animal or man; e.g., Nagamoto et al. 1996). On the other hand, D2 antagonists do show effects in the amphetamine-deficit model in schizophrenia patients, in healthy humans and animals, and in animal models of schizophrenia (e.g., Light et al. 1999; Siegel et al. 2005; During et al. 2014). This indicates that the effects of D2 antagonist are dependent on the model in which it is tested. Although the current study showed improved sensory gating after PDE4 inhibition, further studies in which roflumilast is tested in different models in humans and animals are indicated to further support its potential clinical effects.

Dopamine is not the only neurotransmitter that can affect sensory gating. Other signaling systems, affecting downstream structures capable of exhibiting gating, can show effects in a sensory gating paradigm (see Fig. 1). Both noradrenergic (e.g., Siegel et al. 2005) and cholinergic (e.g., Adler et al. 2001) drugs have shown to affect sensory gating. Especially, the cholinergic system is of interest as a treatment for gating deficits in schizophrenia, as the inhibitory interneurons contain, next to the dopamine D2 receptors, α7 nicotinic acetylcholine receptors which upon activation stimulate GABA (γ-aminobutyric acid) release (e.g., Young and Geyer 2013; see Fig. 1). Via PDE4 expressed in neurons, modulating the inhibitory interneurons, roflumilast could also modulate sensory gating by targeting these different mechanisms. Thus, the current effects may not be explained in terms of an effect on the dopamine system itself but explained by downstream mechanisms in inhibitory interneurons containing D2 receptors that can be affected by PDE4 inhibition (Fig. 1).

This way, different neurobiological mechanisms may underlie the present effects of roflumilast on sensory gating. In general, effects of PDE inhibitors on sensory gating are believed to be induced by targeting PDEs expressed in inhibitory interneurons of the auditory cortex and the thalamic “gate,” frontal inhibitory output neurons or in the interneurons that locally release inhibitory neurotransmitter in any other brain area capable of eliciting sensory gating like the hippocampus. In other words, in the abovementioned brain areas, S1 excites an excitatory neuron, which in turn excites an inhibitory interneuron as well as an excitatory pyramidal neuron (During et al. 2014; see Fig. 1). Activation of the inhibitory interneuron induces release of the inhibitory neurotransmitter GABA. GABA release then causes fast inhibition of the pyramidal neuron via postsynaptic GABA-A receptors. Additionally, GABA released from the inhibitory interneurons induces slow, persistent inhibition of glutamate release from the first excitatory neuron onto the pyramidal neuron via presynaptic GABA-B receptors (Hershman et al. 1995). This persistent inhibition reduces the activity of the pyramidal neuron for up to 8 s. Consequently, if S2 arrives, the ERP amplitude will be reduced.

Hypothetically, targeting any PDE subtype, e.g., PDE4, expressed in the first excitatory neuron’s projections to the inhibitory interneuron as well as PDEs expressed in the inhibitory interneuron itself could enhance output of both neurons when activated by S1. Consequently, the S2-induced amplitude will be further reduced. Note, however, that PDEs expressed in the first excitatory neuron’s projections directly to the pyramidal neuron must not be enhanced since this would increase the response to S1 and therefore positively affect general auditory information processing. Roflumilast enhances sensory gating without exhibiting an effect on S1. Therefore, the effect of roflumilast is more likely to occur in the inhibitory interneurons themselves. PDE4 is indeed relatively highly expressed in brain areas associated with sensory gating (Lakics et al. 2010).

Additionally, PDE4 inhibitors may function like D2 receptor antagonists although it needs to be determined whether this is directly beneficial for sensory gating (Heckman et al. 2017). Taken together, future studies will have to provide more insight into the mechanism by which PDE4 inhibition enhances sensory gating in healthy and pharmacologically impaired volunteers, and eventually patients. Of note, when comparing results, several translational considerations should be taken into account. For example, the site of measurement (intra-cranial in rodents vs scalp in humans), mental state (anesthetized in rodents vs awake in rodents or humans), treatment duration (acute vs chronic), and route of drug administration (mostly intraperitoneal/subcutaneous in rodents vs mostly oral in humans), but also differences in PDE4 expression and pharmacokinetic properties of the drug.

To our knowledge, this is the first study that showed enhanced sensory gating after PDE4 treatment in healthy human subjects. This may indicate that PDE4 inhibition may be relevant for this specific pre-attentive process. Of note, there is also a wealth of data that PDE4 inhibitors also improve memory functions in various animal models (e.g., Jabaris et al. 2015; Vanmierlo et al. 2016). Taken together, this may suggest that PDE4 inhibition may have widespread effects in the brain leading to improvement of different cognitive processes. On basis of the current data, we conclude that a low dose of roflumilast might be considered as a potential treatment for disorders that are characterized by impaired sensory gating.
